# Psychological Treatments and Psychotherapies in the Neurorehabilitation of Pain: Evidences and Recommendations from the Italian Consensus Conference on Pain in Neurorehabilitation

**DOI:** 10.3389/fpsyg.2016.00115

**Published:** 2016-02-19

**Authors:** Gianluca Castelnuovo, Emanuele M. Giusti, Gian Mauro Manzoni, Donatella Saviola, Arianna Gatti, Samantha Gabrielli, Marco Lacerenza, Giada Pietrabissa, Roberto Cattivelli, Chiara A. M. Spatola, Stefania Corti, Margherita Novelli, Valentina Villa, Andrea Cottini, Carlo Lai, Francesco Pagnini, Lorys Castelli, Mario Tavola, Riccardo Torta, Marco Arreghini, Loredana Zanini, Amelia Brunani, Paolo Capodaglio, Guido E. D'Aniello, Federica Scarpina, Andrea Brioschi, Lorenzo Priano, Alessandro Mauro, Giuseppe Riva, Claudia Repetto, Camillo Regalia, Enrico Molinari, Paolo Notaro, Stefano Paolucci, Giorgio Sandrini, Susan G. Simpson, Brenda Wiederhold, Stefano Tamburin

**Affiliations:** ^1^Psychology Research Laboratory, Istituto Auxologico Italiano IRCCS, San Giuseppe HospitalVerbania, Italy; ^2^Department of Psychology, Catholic University of MilanMilan, Italy; ^3^Faculty of Psychology, eCampus UniversityNovedrate (Como), Italy; ^4^Cardinal Ferrari Rehabilitation Center, Santo Stefano Rehabilitation IstituteFontanellato, Italy; ^5^Private PracticeParma, Italy; ^6^Casa di Cura San Pio X S.r.l., HUMANITASMilan, Italy; ^7^IRCCS Galeazzi Orthopedic InstituteMilan, Italy; ^8^Department of Dynamic and Clinical PsychologySapienza University of Rome, Italy; ^9^Department of Psychology, Harvard UniversityCambridge, MA, USA; ^10^Department of Psychology, University of TurinTurin, Italy; ^11^Villa Scassi HospitalGenova, Italy; ^12^Department of Neuroscience “Rita Levi Montalcini”University of Turin, Italy; ^13^Rehabilitation Unit, Istituto Auxologico Italiano IRCCS, San Giuseppe HospitalVerbania, Italy; ^14^Department of Neurology and Neurorehabilitation, Istituto Auxologico Italiano IRCCS, San Giuseppe HospitalVerbania, Italy; ^15^“Pain Center II Level - Department of Surgery” - ASST Grande Ospedale Metropolitano NiguardaMilano, Italy; ^16^Fondazione Santa Lucia IRCCSRome, Italy; ^17^Department of Brain and Behavioral Sciences, C. Mondino National Neurological Institute, University of PaviaPavia, Italy; ^18^School of Psychology, Social Work and Social PolicyUniversity of South Australia, Australia; ^19^Virtual Reality Medical InstituteBrussels, Belgium; ^20^Department of Neurological and Movement Sciences, University of VeronaVerona, Italy

**Keywords:** psychological treatments, psychotherapy, neurological rehabilitation, chronic pain, pain, clinical psychology, health psychology

## Abstract

**Background:** It is increasingly recognized that treating pain is crucial for effective care within neurological rehabilitation in the setting of the neurological rehabilitation. The Italian Consensus Conference on Pain in Neurorehabilitation was constituted with the purpose identifying best practices for us in this context. Along with drug therapies and physical interventions, psychological treatments have been proven to be some of the most valuable tools that can be used within a multidisciplinary approach for fostering a reduction in pain intensity. However, there is a need to elucidate what forms of psychotherapy could be effectively matched with the specific pathologies that are typically addressed by neurorehabilitation teams.

**Objectives:** To extensively assess the available evidence which supports the use of psychological therapies for pain reduction in neurological diseases.

**Methods:** A systematic review of the studies evaluating the effect of psychotherapies on pain intensity in neurological disorders was performed through an electronic search using PUBMED, EMBASE, and the Cochrane Database of Systematic Reviews. Based on the level of evidence of the included studies, recommendations were outlined separately for the different conditions.

**Results:** The literature search yielded 2352 results and the final database included 400 articles. The overall strength of the recommendations was medium/low. The different forms of psychological interventions, including Cognitive—Behavioral Therapy, cognitive or behavioral techniques, Mindfulness, hypnosis, Acceptance and Commitment Therapy (ACT), Brief Interpersonal Therapy, virtual reality interventions, various forms of biofeedback and mirror therapy were found to be effective for pain reduction in pathologies such as musculoskeletal pain, fibromyalgia, Complex Regional Pain Syndrome, Central Post—Stroke pain, Phantom Limb Pain, pain secondary to Spinal Cord Injury, multiple sclerosis and other debilitating syndromes, diabetic neuropathy, Medically Unexplained Symptoms, migraine and headache.

**Conclusions:** Psychological interventions and psychotherapies are safe and effective treatments that can be used within an integrated approach for patients undergoing neurological rehabilitation for pain. The different interventions can be specifically selected depending on the disease being treated. A table of evidence and recommendations from the Italian Consensus Conference on Pain in Neurorehabilitation is also provided in the final part of the paper.

## Introduction

Pain is frequent in the setting of neurorehabilitation. Most patients undergoing rehabilitation for neurological diseases complain of pain. Both pain and side effects of the drugs used to provide relief from pain from pain may interfere or have a negative effect within the rehabilitation process (Gallagher, 2005; Pongparadee et al., 2012; Singh et al., 2013; Desai et al., 2015). Moreover, pharmacological therapies are effective only in a minority of patients with neuropathic pain (NP) or pain associated with neurological conditions. To date, there are no specific guidelines on the treatment of pain in neurorehabilitation. The Italian Consensus Conference on Pain in Neurorehabilitation (ICCPN) was established in October 2012 and aims to collect and review the evidence and to offer updated conclusions on the treatment of pain in this setting. The ICCPN is composed of a multidisciplinary board involving physicians, psychologists, physiotherapists and other medical and clinical experts. An effective pain treatment in the neurorehabilitation setting requires the contribution of all these specialists as it is now clear that biological and psychological aspects influence each other in a complex way to generate, maintain and modify the patient's experience of pain (Castelnuovo, 2010a,b, 2013; Chang et al., 2014; Gallien et al., 2014; Hussain and Erdek, 2014; Simons et al., 2014; Zheng et al., 2014; Cragg et al., 2015; Durand et al., 2015; Allegri et al., 2016). Psychological therapies play an important role in the multidisciplinary treatment of pain in the neurorehabilitation setting because of their efficacy and the general absence of side effects. The experience of pain, in particular when chronic, is often associated with a general discomfort that can foster conditions of anxiety, depression and insomnia, consistently affecting the quality of life of the patient. Psychotherapies, through different mechanisms, act on three levels:

- treatment of comorbid conditions (e.g., depression and anxiety);
- improvement in psychological issues that, if not treated, can contribute by maintaining the painful condition;
- reduction in perceived pain through activation of descending inhibitory control systems.

These three aspects are strongly interrelated. The painful experience is often worsened by maladaptive changes in physiological systems, such as the sleep—wake processes and the stress reaction systems, and in psychological processes, such as cognition, mood, and motivation, consequently affecting the behavior of the patient (Wiech and Tracey, 2013; Simons et al., 2014; Tamburin et al., 2014). Conversely, the different forms of psychological interventions may have positive effects on these domains, both triggering a readjustment of the physiological processes (e.g., biofeedback and virtual reality interventions) and\or tackling maladaptive thoughts and attitudes about pain, and\or leading to new pain coping strategies (Turk et al., 2010; Sturgeon, 2014).

It should be noted that psychological treatments in general can lead to a mild or moderate reduction in pain intensity. That is a significant result, as different pain diseases are difficult to treat with drugs and because neuropathic pain responds to the current pharmacological therapies at most in 30–40% of cases (Magrinelli et al., 2013). Nevertheless, drug therapies and non-drug therapies should not be considered as mutually exclusive: different treatments might be integrated with each other and the simultaneous action on different aspects of the disease, conducted by professionals belonging to different disciplines, ensures greater effectiveness of care (Guzman et al., 2006; Turk et al., 2010).

The ICCPN systematically reviewed the evidence regarding the role of psychotherapy in the neurorehabilitation setting. A search of all the research reports, systematic reviews or meta-analyses which evaluated psychological therapies, addressed neurological conditions and considered pain as an outcome was performed in PUBMED, EMBASE, and the Cochrane Database of Systematic Reviews. Accordingly, keywords included “pain,” “neurological diseases” the names of specific neurological conditions, “psychological therapies” the names of specific psychotherapies (see Box 1 for search strings and Box 2 for the main psychotherapies considered). The presence of an evaluation of at least one psychological treatment for pain intensity in at least one neurological condition was also used as the inclusion criterion in the subsequent steps. The search was conducted on October 2013 and yielded 2038 articles, two updates were then performed on July 2014 and January 2015 restricting the results to the 2013–2014 period, yielding respectively 225 and 89 additional articles. The selected research reports were then assessed on whether they met the inclusion criterion examining their abstract or, if needed, their full text. During this phase their bibliographies were scanned and other relevant articles were included. The final database was composed of 400 articles. The studies were rated good, fair, or poor quality following a checklist specifically built to assess the number of subjects included, the dropout rate, the risk of bias (i.e., assessment of potential confounders) and the presence of blinding procedures. Reviews and meta-analyses were rated according to the comprehensiveness of the literature search and the assessment of the risk of bias. The level of evidence was then assigned to each article following an adaptation of the SIGN grading system (Harbour and Miller, 2001; Table 1) and the recommendations were formulated accordingly (Table 2), considering pain intensity reduction as the only outcome.

Box 1 **Search strings.**

**Table:** 

**Pubmed:**	**Embase:**	**Cochrane database of systematic reviews:**
(PDN OR “neuropathy” OR “brain injury” OR “multiple sclerosis” OR stroke OR “cerebral palsy” OR “post-polio syndrome” OR parkinson OR guillain-barre OR “nervous system diseases”[MeSH]) AND (rehabilitation^*^ OR neurorehabilitation OR therapy) AND pain[Title/abstract] AND (psychotherapy[title/abstract] OR “cognitive therapy”[title/abstract] OR “group therapy”[Title/abstract] OR “family therapy”[Title/abstract] OR mindfulness[Title/abstract] OR biofeedback[Title/abstract] OR hypnosis[title/abstract] OR “cognitive-behavioral”[title/abstract] OR “psychodynamic”[title/abstract] OR “brief therapy”[title/abstract] OR “autogenic training”[title/abstract] OR “psychological treatment^*^”[title/abstract] OR “virtual reality”[title/abstract]) AND (trial OR review OR RCT OR “case reports”[publication type] OR “clinical trial”[publication type] OR “comparative study”[publication type] OR “meta-analysis”[publication type] OR “cohort study^*^” OR “case-control” OR “efficacy” OR “pain reduction” OR “pain management” OR “panel study”)	neurologic disease'/exp OR ‘neurologic disease’ AND pain:ab,ti AND (psychotherapy:ab,ti OR ‘cognitive therapy’:ab,ti OR ‘behavioral therapy’:ab,ti OR ‘cognitive-behavioral’:ab,ti OR mindfulness:ab,ti OR hypnosis:ab,ti OR ‘brief therapy’:ab,ti OR ‘psychodynamic therapy’:ab,ti OR ‘acceptance therapy’:ab,ti OR ‘autogenic training’:ab,ti OR biofeedback:ab,ti OR ‘virtual reality’:ab,ti OR ‘psychological treatment’:ab,ti)	#1: MeSH descriptor: [Nervous System Diseases];
		#2: pain and (psychotherapy or “cognitive therapy” or “behavioral therapy” or “cognitive-behavioral therapy” or “hypnosis” or biofeedback or “psychodinamic” or “brief therapy” or “acceptance therapy” or “family therapy” or “virtual reality”)
		#3: #1 and #2

Box 2 **Definitions of the main psychotherapeutic approaches reported in the article (using common and popular sources such as wikipedia and other informative websites - january 2016).**

**Psychological interventions:** actions performed to bring about change in people. A wide range of intervention strategies exist and they are directed toward various types of issues. Most generally, it means any activities used to modify behavior, emotional state, or feelings. Psychological interventions have many different applications and the most common use is for the treatment of mental disorders, most commonly using psychotherapy.

**Psychotherapy:** psychotherapy is the use of psychological methods, particularly when based on regular personal interaction, to help a person change and overcome problems in desired ways. Psychotherapy aims to increase each individual's well-being and mental health, to resolve or mitigate troublesome behaviors, beliefs, compulsions, thoughts, or emotions, and to improve relationships and social functioning.

**Acceptance and Commitment Therapy (ACT)**: a type of psychological intervention that focuses on the development of psychological flexibility, or the ability to contact the present moment and accept negative thoughts without judgment.

**Biofeedback intervention**: a treatment technique in which people are trained to improve their health by using signals from their own bodies.

**Cognitive Behavioral Therapy (CBT)**: a broad range of psychotherapies that aim to help clients overcome dysfunctional thought patterns and behavioral patterns.

**Eye Movement Desensitization and Reprocessing therapy (EMDR)**: a psychotherapy developed by Francine Shapiro that reduces the long-lasting effects of distressing memories by developing more adaptive coping mechanisms.

**Hypnosis-Hypnotic therapies**: a form of psychotherapy used to create subconscious change in a patient in the form of new responses, thoughts, attitudes, behaviors or feelings.

**Mindfulness based interventions**: programs developed by Jon Kabat-Zinn that include mindfulness meditation and yoga. They are based on the concept of mindfulness, or being fully engaged in the present moment rather than worrying about past or future events, an ancient concept in Buddhist psychology.

**Mirror therapy**: an approach originally developed for the relief of Phantom Limb Pain that uses a mirror box (which is a box with two mirrors in the center - one facing each way) and draws on the principle of visual feedback.

**Psychodynamic therapy**: a psychotherapy that focuses on unconscious processes as they are manifested in a person's present behavior. The goals are to develop the client's self-awareness and understanding of the influence of the past on present behavior.

**Relaxation training-tecnique:** any method, process, procedure, or activity that helps a person to relax; to attain a state of increased calmness; or otherwise reduce levels of pain, anxiety, stress or anger.

**Table 1:** **Levels of evidence (Harbour and Miller, 2001)**.

**Levels of evidence**	**Type of evidence**
1++	High quality meta-analyses, systematic reviews of RCTs, or RCTs with a very low risk of bias
1+	Well-conducted meta-analyses, systematic reviews, or RCTs with a low risk of bias
1−	Meta-analyses, systematic reviews, or RCTs with a high risk of bias
2++	High quality systematic reviews of case control or cohort or studies; high quality case control or cohort studies with a very low risk of confounding or bias and a high probability that the relationship is causal
2+	Well-conducted case control or cohort studies with a low risk of confounding or bias and a moderate probability that the relationship is causal
2−	Case control or cohort studies with a high risk of confounding or bias and a significant risk that the relationship is not causal
3	Non-analytic studies, e.g., case reports, case series
4	Expert opinion

**Table 2:** **Grades of recommendations (Harbour and Miller, 2001)**.

**Grades of recommendations**	**Evidence**
A	At least one meta-analysis, systematic review, or RCT rated as 1++, and directly applicable to the target population; or a body of evidence consisting principally of studies rated as 1+, directly applicable to the target population, and demonstrating overall consistency of results
B	A body of evidence including studies rated as 2++, directly applicable to the target population, and demonstrating overall consistency of results; or extrapolated evidence from studies rated as 1++ or 1+
C	A body of evidence including studies rated as 2+, directly applicable to the target population and demonstrating overall consistency of results; or extrapolated evidence from studies rated as 2++
D	Evidence level 3 or 4; or extrapolated evidence from studies rated as 2+
GPP	Recommended best practice based on the clinical experience of the guideline development group

## Evidences and discussion

The analysis of collected data suggests that psychological therapies are highly indicated both for the treatment of painful conditions and for the treatment of pain related to several neurological diseases (Table 3). The reviews and meta-analyses conducted to evaluate the effectiveness of different forms of psychotherapy across several disorders, albeit with different levels of experimental evidence, confirmed that psychological interventions can improve the experience of patients, both in adults (Raine et al., 2002; Astin et al., 2003; Williams et al., 2012) and in children and adolescents (Eccleston et al., 2014; Fisher et al., 2014). Similar results were reported by the reviews and meta-analyses that evaluated the effect of psychotherapy as addressed to the treatment of specific pain disorders such as low back pain (Nielson and Weir, 2001; Chou and Huffman, 2007; Hoffman et al., 2007), fibromyalgia (Lami et al., 2013), tension-type Headache (TTH) and migraine (Andrasik, 2007), pain associated with rheumatoid arthritis (Astin et al., 2002; Knittle et al., 2010), chronic abdominal pain in adolescents (Sprenger et al., 2011), and chronic orofacial pain (Aggarwal et al., 2011), although in the latter instance the authors indicate a high risk of biases in their conclusions. The study on the impact of the different forms of psychotherapy on phantom limb pain is also promising (Moura et al., 2012; Niraj and Niraj, 2014) although, in this case, more research is needed to validate the effects.

**Table 3:** **Summary table of evidence and recommendations from the Italian Consensus Conference on Pain in Neurorehabilitation**.

Psychological therapies are highly recommended for the treatment of painful conditions and for the treatment of pain associated with various neurological diseases. Psychological therapies act on three levels: treatment of psychopathological comorbidities, reduction in perceived pain, improvement in the psychological aspects that contribute to maintain the pain. Most interventions are more effective and may enhance the outcomes of pharmacological and physical therapies if they are included in multidisciplinary treatments (GPP).
Recommended interventions for the whole of the chronic pain syndromes with heterogeneous physiopathology are: Mindfulness (A), Cognitive Behavioral Therapy (CBT), either conducted in individual, group setting or administered via computer (B), multidisciplinary interventions (B) and hypnosis (B).
Acute musculoskeletal pain is rarely treated with psychological treatments due to its favorable prognosis and waiting-list issues. However, CBT is considered effective for this condition (C). For chronic musculoskeletal pain multidisciplinary interventions (A), CBT in individual setting (B) or in group setting (A) and educational and behavioral interventions (B) are recommended. Also biofeedback may be used (GPP).
Telephone – delivered CBT can be used for the treatment of Chronic Widespread Pain (GPP). With regards to fibromyalgia, CBT (A), educational and behavioral interventions for the management of the disease in daily life (B), multidisciplinary interventions (B), mindfulness (C), electromyographic biofeedback and neurofeedback (GPP), and Acceptance and Commitment Therapy conducted in group setting (GPP) have been proven effective. CBT is recommended for the treatment of juvenile fibromyalgia (C). It is not possible to give recommendations for the treatment of pain associated with Chronic Fatigue Syndrome.
Complex Regional Pain Syndrome type I due to stroke or injury can be treated with motor imagery interventions (GPP) or with mirror therapy (if the pathology is due to stroke) (GPP). Early evidence may support the use of cognitive interventions or CBT associated with physical therapies (e.g. TENS, massages) (GPP). An interdisciplinary approach that includes physical, occupational and cognitive-behavioral interventions can be used for the treatment of CRPS-I in pediatric patients (GPP). No recommendation can be made with regards to CRPS-II.
For Central Post-Stroke Pain, mirror therapy and immersive virtual reality interventions are recommended (GPP).
For the treatment of Phantom Limb Pain, hypnosis, mirror therapy, immersive virtual reality interventions and EMDR are recommended (D).
Neuropathic pain secondary to spinal cord injury is difficult to treat; therefore, it is necessary to use multidisciplinary interventions on its different symptoms (GPP). The most effective approach can be the use of hypnosis (D) or virtual reality protocols in particular if associated with hypnosis or transcranial Direct Current Stimulation (D).
For chronic pain associated with multiple sclerosis, hypnosis (D) and virtual reality interventions (D) are recommended.
Hypnosis is recommended for patients suffering from pain associated with Amyotrophic Lateral Sclerosis, Parkinson's Disease, Guillain-Barré syndrome, HIV and Post – Polio Syndrome (GPP).
For the treatment of diabetic neuropathy and neuropathic pain associated with cancer or HIV, CBT may be used (GPP).
Electromyographic biofeedback interventions or protocols that combine relaxation techniques and biofeedback are effective in the treatment of pain associated with cervical dystonia (D), cerebral palsy, focal hand dystonia and postherpetic neuralgia (GPP).
It is possible to give only a weak recommendation for the treatment of chronic pain associated with Rheumatoid Arthritis. Early evidences support the use of hypnosis (GPP), interventions based on patient's education or on relaxation (GPP) and interventions based on meditation (GPP). CBT is effective on various psychological aspects associated with pain in Rheumatoid Arthritis but not on pain intensity. A multidisciplinary intervention is recommended for the treatment of Ehlers-Danlos Syndrome (GPP) and biofeedback may be used for the care of people affected by systemic lupus erythematosus (GPP).
For the treatment of chronic Tension-type Headache and migraine, electromyographic, thermal and electrogalvanic biofeedback interventions (A) in addition to autogenic training, relaxation training (B), hypnosis (C), and biofeedback intervention combined with virtual reality (GPP) are recommended. A very low recommendation can be given for hypnosis for the treatment of pain due to post-concussion syndrome (GPP) and to mindfulness therapies for the treatment of post-traumatic headache (GPP).
Burning Mouth Syndrome and facial pain can be treated with CBT or psychodynamic therapy combined with pharmacological interventions (GPP).
For temporomandibular disorders, Brief CBT or CBT conducted in group settings integrated with pharmacological interventions or hypnosis (B), and hypnosis (C) are recommended.
Medically Unexplained Symptoms and somatoform disorders can be treated with CBT conducted in group setting or as a part of a multidimensional approach that combines medication and psychotherapy, as well as Brief Dynamic Interpersonal Therapy (GPP).
There is preliminary evidence supporting the effectiveness of cognitive – behavioral therapies on chronic abdominal pain in children (C).

A number of studies evaluated the effects of psychological therapies for chronic pain, grouping together under this category various forms of persistent pain with heterogeneous pathophysiology, including musculoskeletal nociceptive pain, pain secondary to osteoarthritis or rheumatoid arthritis, fibromyalgia, chronic headache, and migraine.

For these conditions the following approaches are recommended:

- *Mindfulness* interventions (Grade of recommendation: A) (Grossman et al., 2004; Gardner-Nix et al., 2008; Teixeira, 2008; Rosenzweig et al., 2010; Chiesa and Serretti, 2011; Veehof et al., 2011; Wong et al., 2011; Lakhan and Schofield, 2013)
- Cognitive Behavioral Therapy (CBT), both in individual setting (Grade of recommendation: B) (McCarberg and Wolf, 1999; Morley et al., 1999; Lunde et al., 2009), group setting (Grade of recommendation: B) (Moore and Chaney, 1985; Ersek et al., 2003; Elomaa et al., 2009; Thorn et al., 2011), and internet—based, both for adults (Grade of recommendation: B) (Macea et al., 2010; Ruehlman et al., 2012; Nevedal et al., 2013), and for pediatric patients (Grade of recommendation: B) (Hicks et al., 2006; Palermo et al., 2009).
- Hypnotic therapies: systematic reviews (Hawkins, 2001; Elkins et al., 2007), while stressing that there are many methodologically weak studies in literature, support their analgesic power, and this effect has been confirmed by a meta-analysis (Montgomery et al., 2000) (Grade of recommendation: B).
- Virtual reality: VR-based distraction interventions have been used in acute pain management for over a decade and a systematic review suggests its use for clinicians who work with a variety of pain problems (Malloy and Milling, 2010). While sense of presence influences the effectiveness of VR as a distraction tool, anxiety as well as positive emotions directly affect the experience of pain (Triberti et al., 2014). However the use of VR with chronic pain is still in its infancy and only a few controlled trials are available (Hua et al., 2015; Roosink et al., 2015) (Grade of recommendation: D).
- The techniques of self-management for chronic have been evaluated by a single randomized controlled trial (Kroenke et al., 2009) and, therefore, they are still to be assessed extensively. Also Acceptance and Commitment Therapy (ACT), an extension of CBT (Vowles et al., 2014), cannot be recommended for the treatment of chronic pain. Indeed, not all of the studies published so far have found empirical evidence to support the effectiveness of specific psychological therapies on pain intensity, when conducted in individual setting, group setting, or administered via computer (Vowles and McCracken, 2008; Wicksell et al., 2009; Thorsell et al., 2011; Wetherell et al., 2011; Buhrman et al., 2013; McCracken et al., 2013).

According to the biopsychosocial approach (Engel, 1977), all these treatments have a higher effectiveness when included into multidimensional and multidisciplinary interventions, and their efficacy is even greater than the pharmacological therapies or physical therapies alone (Grade of recommendation: B) (Lipchik et al., 1993; Mattenklodt et al., 2008; Samwel et al., 2009; Pieh et al., 2012; Gagnon et al., 2013).

Taking into consideration specific pain conditions, psychological therapies have the potential to play a major role in the treatment of acute and chronic musculoskeletal pain. The majority of international guidelines agree about the importance of psychological approaches for treating a single episode of back pain (Koes et al., 2001, 2010) and, although there is strong evidence that preventing acute pain from becoming chronic is cost-effective (Waddell and Burton, 2001), this condition is seldom treated with psychotherapies, mostly for waiting-list reasons. Therefore, few psychological interventions have been evaluated for back pain in its early stages. However, research supports that CBT or treatments that feature cognitive techniques are able to prevent its evolution to chronicity, the increase in use of health care resources and days of absence from work, as well as modestly reducing the intensity of the perceived pain (Grade of recommendation: C) (Hasenbring et al., 1999; Linton and Andersson, 2000; Sullivan et al., 2006; Slater et al., 2009). For chronic musculoskeletal pain, interventions that appear to be more effective are the multidisciplinary and multidimensional ones (Grade of recommendation: A) (Guzman et al., 2006; Kääpä et al., 2006; Mangels et al., 2009; Mannion et al., 2013; Monticone et al., 2013; Kamper et al., 2014). CBT, both conducted in individual settings (Grade of recommendation: B, these studies are not always methodologically perfect and the impact on pain is still quite low) (Turner and Jensen, 1993; Turner, 1996; Rose et al., 1997; Smeets et al., 2006; Trapp et al., 2009; Glombiewski et al., 2010a; Taloyan et al., 2013) and group settings (Grade of recommendation: A) (Turner et al., 1990; Turner and Jensen, 1993; Newton-John et al., 1995; Basler et al., 1997; Rose et al., 1997; Haldorsen et al., 1998; Linton and Ryberg, 2001; Linton and Nordin, 2006; Lamb et al., 2010a,b), and educational and behavioral interventions (Grade of recommendation: B) (Tavafian et al., 2007; Brox et al., 2008; Henschke et al., 2010; van Middelkoop et al., 2011) are also highly recommended. It must be noted that the efficacy of cognitive-behavioral therapies are consistent across both individual and group settings (Rose et al., 1997) and that all of the interventions previously listed are also effective are also effective in the context of patients planning early retirement as a result of pain or a highly disabling condition, both of which are frequently perceived as obstacles to improvement (Trapp et al., 2009).

It is not possible to conclusively determine the effectiveness of electromyographic, postural and respiratory biofeedback interventions for chronic musculoskeletal pain. In fact, the methodological quality of the studies addressing this issue is often insufficient and the two best conducted trials (Ehrenborg and Archenholtz, 2010; Kapitza et al., 2010) do not support a specific effect of these therapies; however, there are some data in support of its analgesic potential (Flor and Birbaumer, 1993; Magnusson et al., 2008; Glombiewski et al., 2010a; Hallman et al., 2011; Ma et al., 2011). The heterogeneity of the results may be due to differences in the methodological quality of the studies and in the interventions examined. It is also possible that the short-term effect of biofeedback is similar to the early outcome of cognitive behavioral therapies, while the long-term benefits of the former could be greater (Flor and Birbaumer, 1993; Glombiewski et al., 2010a). Further studies will shed light on the subject (Grade of recommendation: GPP).

Psychological therapies can be a valuable resource in the treatment of Chronic Widespread Pain (CWP) and fibromyalgia (FM), syndromes that are notoriously difficult to manage with a pharmacological approach. Telephone-delivered CBT has been proven to be an effective intervention for CWP, although there is a need for further research to support this conclusion (Grade of recommendation: GPP) (McBeth et al., 2012). There is general agreement on the fact that most psychological therapies can lead to clinically significant improvements in the experience of patients suffering from FM as these can prevent and treat depressive symptoms often associated with the condition, promote the management of insomnia and fatigue and reduce the impact of psychological factors related to the pain. If the criterion is the reduction of perceived pain, there are differences between the various interventions. CBT is effective both in the treatment of related symptoms and for the reduction in the intensity of perceived pain (Grade of recommendation: A) (Rossy et al., 1999; Bernardy et al., 2010; Glombiewski et al., 2010b; Gritzner et al., 2012; Clauw, 2014). Psycho-educational and behavioral interventions, aimed at helping the patient to improve the management of the disease in his daily life, appear also to be effective in reducing perceived pain (Grade of recommendation: B) (Burckhardt et al., 1994; Nicassio et al., 1997; Thieme et al., 2003, 2006; Hammond and Freeman, 2006) as well as multidisciplinary treatments (Grade of recommendation: B) (Lemstra and Olszynski, 2005; Lera et al., 2009; Lange et al., 2011; Vincent et al., 2013).

Studies on the effectiveness of mindfulness-based interventions, that seem to be a very promising resource in the treatment of FM (Grade of recommendation: C) (Grossman et al., 2007; Schmidt et al., 2011; Lauche et al., 2013; Shaheen, 2014), and ACT in a group setting (Grade of recommendation: GPP) (Luciano et al., 2014) are still conflicting. Electromyographic biofeedback and neurofeedback interventions may also be used, even if further studies are needed to support the effectiveness of these therapies, since the best study conducted so far did not identify any significant effect (Santen et al., 2002). The class of recommendation for these interventions is low, although there are two randomized controlled trials and two case series that support their role in pain management (Grade of recommendation: GPP) (Mur et al., 1999; Babu et al., 2007; Hassett et al., 2007; Kayiran et al., 2010). Finally, CBT is effective for juvenile fibromyalgia (Grade of recommendation: C) (Kashikar-Zuck et al., 2005, 2012; Degotardi et al., 2006).

Conversely, it is not possible to make a recommendation for the treatment of pain associated with Chronic Fatigue Syndrome. Research on the topic, mainly focused on CBT conducted in individual or group settings, gave mixed results (Stulemeijer et al., 2004; Núñez et al., 2011; Bloot et al., 2015) and the underlying rationale has been questioned (Twisk and Maes, 2009). Therefore, further studies are needed.

Several imagery or visual feedback interventions can be used in the care of patients with a diagnosis of Complex Regional Pain Syndrome type 1 (CRPS-I), although further studies are needed to confirm their effectiveness. In particular, motor imagery interventions are recommended for CRPS-I due to injury or stroke (Grade of recommendation: GPP) (Moseley, 2004, 2005, 2006; O'Connell et al., 2013) and mirror therapy is recommended for CRPS-I due to stroke (Grade of recommendation: GPP) (Cacchio et al., 2009; Sato et al., 2010; O'Connell et al., 2013). It is to be noted that the first reported attempts to use graded motor imagery in clinical practice for this condition did not yield significant result, perhaps due to the lower control of the therapeutic procedures (Johnson et al., 2012).

Preliminary data indicate that cognitive interventions based on graded exposure and CBT associated with physical therapies (TENS, massages, etc.) could also be used (Grade of recommendation: GPP): (Lee et al., 2002; De Jong and Vlaeyen, 2005). In the treatment of CRPS-I an interdisciplinary approach that combines physical, occupational, and cognitive-behavioral interventions may lead to clinically significant results with pediatric patients (Grade of recommendation: GPP) (Patterson, 2011; Logan et al., 2012). There is insufficient evidence to draw conclusions about the efficacy of psychological interventions for CRPS-II (O'Connell et al., 2013).

Among the other conditions associated with neuropathic pain, there is early evidence that Central Post-stroke Pain may be effectively treated with mirror therapy and with immersive virtual reality interventions (Grade of recommendation: GPP) (Rodriguez et al., 2011; Thieme et al., 2012).

Phantom limb pain can be addressed with different psychological treatments. Early findings, mainly based on case reports, support the use of hypnosis (Grade of recommendation: D) (Rosén et al., 2001; Oakley et al., 2002; Bamford, 2006; Niraj and Niraj, 2014), biofeedback interventions (Grade of recommendation: D) (Belleggia and Birbaumer, 2001; Flor et al., 2001; Harden et al., 2005), Eye Movement Desensitization and Reprocessing therapy (EMDR) (Grade of recommendation: D) (Schneider et al., 2008; de Roos et al., 2010), and mirror therapy (Grade of recommendation: D) (Brodie et al., 2007; Chan et al., 2007; Murray et al., 2007; Mercier and Sirigu, 2009; Seidel et al., 2011) in the treatment of this condition, although there is no general agreement on the effectiveness of the latter.

Neuropathic pain is one of the most debilitating complications of spinal cord injury and since the underlying mechanisms are only partly understood, it is difficult to treat (Wrigley et al., 2009; Defrates and Cook, 2011). Therefore, multidisciplinary neurorehabilitation interventions acting simultaneously on different symptoms are needed (Grade of recommendation: GPP) (Heutink et al., 2014). So far, hypnotic treatments (Grade of recommendation: D) (Jensen and Barber, 2000; Jensen et al., 2009c, 2013; the recommendation is limited as all three studies were conducted by the same author) and virtual reality protocols (that are more effective when associated with hypnosis or transcranial Direct Current Stimulation (tDCS)), (grade of recommendation: D) (Moseley, 2007; Oneal et al., 2008; Soler et al., 2010; Villiger et al., 2012, 2013; Boldt et al., 2014) have been evaluated.

Hypnosis might be an effective intervention in the case of chronic pain associated with Multiple Sclerosis (Grade of recommendation: D) (Dane, 1996; Jensen et al., 2009a,b, 2011; Tierno et al., 2014). Preliminary evidence supports the use of hypnosis in the treatment of pain in various neurological conditions, including Amyotrophic Lateral Sclerosis (Palmieri et al., 2012; Kleinbub et al., 2015), Parkinson's Disease (Elkins et al., 2013), Guillain-Barré Syndrome (Fowler and Falkner, 1992), neuropathic pain due to HIV (Dorfman et al., 2013), and Post-Polio Syndrome (Hammond, 1991) (Grade of recommendation: GPP). The role of CBT in the treatment of pain due to diabetic neuropathy (Otis et al., 2013), neuropathic cancer pain (Steggles, 2009), and neuropathic HIV pain (Evans et al., 2003) is under evaluation (Grade of recommendation: GPP).

Electromyographic biofeedback interventions and the use of relaxation techniques that feature biofeedback appear to be promising for the treatment of chronic pain associated with Cervical Dystonia (Grade of recommendation: D) (Smania et al., 2003; Mueller and Wissel, 2010; De Pauw et al., 2014), Cerebral Palsy (Engel et al., 2004), Focal Hand Dystonia (Deepak and Behari, 1999), and Postherpetic Neuralgia (Ing, 2007) (Grade of recommendation: GPP).

It is possible to make only weak recommendations regarding the treatment of chronic pain associated with Rheumatoid Arthritis. Although it is established that different psychological therapies can have a short-term effect on the intensity of pain, related studies are very heterogeneous and the statistical power of analysis conducted is often low (Astin et al., 2002). Early evidence supports the use of hypnosis (Horton-Hausknecht et al., 2000), interventions based on relaxation or arthritis education (Barsky et al., 2010), and an Internal Family Systems—based intervention similar to mindfulness therapy (Shadick et al., 2013) (Grade of recommendation: GPP). The trials that evaluated CBT agree about its positive effects on different physical and psychological aspects but reported non—significant effects on pain intensity (Leibing et al., 1999; Sharpe et al., 2001; Evers et al., 2002; Zautra et al., 2008; Sharpe and Schrieber, 2012). With regards to other forms of rheumatic diseases, multidisciplinary treatments may be recommended for Ehlers-Danlos Syndrome (Bathen et al., 2013) (Grade of recommendation: GPP) and biofeedback therapy may be indicated for the treatment of systemic lupus erythematosus (Greco et al., 2004).

Various biofeedback modalities might be used for the treatment of TTH and migraine patients. In particular electromyographic, thermal, and electrogalvanic biofeedback interventions have been proven effective both with adults and pediatric patients when included in multidimensional programs, in addition to cognitive-behavioral therapies, or administered as single treatments (Grade of recommendation: A) (Falkenstein et al., 1985; Fentress et al., 1986; Grazzi et al., 1990; Allen and McKeen, 1991; Allen and Shriver, 1998; Gatti et al., 2002; Scharff et al., 2002; Vasudeva et al., 2003; Nestoriuc and Martin, 2007; Nestoriuc et al., 2008; Mullally et al., 2009; Bembalgi and Naik, 2012, 2013; Magnoux et al., 2012; Gomez et al., 2013; Sanchez et al., 2013). The meta-analysis conducted by Nestoriuc et al. (2008), stated that biofeedback interventions can be effective for the treatment of TTH and migraine. Autogenic training, relaxation training (Grade of recommendation: B) (Janssen and Neutgens, 1986; Vandyck et al., 1991; Spinhoven et al., 1992; Ter Kuile et al., 1994; Stetter and Kupper, 2002; Pickering et al., 2012) and hypnosis (Grade of recommendation: C) (Berlin et al., 1985; Zitman et al., 1992; Hammond, 2007) may be used on adult patients. In addition, preliminary data support the effectiveness of treatments that involve a combination of biofeedback and virtual reality (Grade of recommendation: GPP) (Shiri et al., 2013). Research to support the effect mindfulness-based interventions on post-traumatic headache is still ongoing (Grade of recommendation: GPP) (Bédard et al., 2012). Finally, one study supports the use of hypnosis for the treatment of pain in post—concussion syndrome (GPP) (Dilks and Bourassa, 2012).

Psychotherapies have proven effective in the treatment of Burning Mouth Syndrome, facial pain, and temporomandibular disorders. Regarding the first, CBT, conducted in individual and group settings, appears to be promising (Grade of recommendation: GPP) (Bergdahl et al., 1995; Miziara et al., 2009) as well as psychodynamic interventions combined with pharmacological interventions (Grade of recommendation: GPP) (Femiano et al., 2004). Brief CBT, CBT conducted in group setting, or CBT integrated with pharmacological interventions or hypnosis are recommended for the treatment of temporomandibular disorders (Grade of recommendation: B) (Dworkin et al., 2002; Turner et al., 2006; Ferrando et al., 2012; Wang et al., 2012). Finally, hypnosis may be used in the treatment of these disorders as well as in the care of people with Persistent Idiopathic Facial Pain (Grade of recommendation: C) (Simon and Lewis, 2000; Abrahamsen et al., 2008, 2011).

Psychotherapy interventions are recommended in the treatment of patients with Medically Unexplained Symptoms and Somatoform Disorders to reduce the pain component. So far, CBT, conducted in group settings or included in a personalized multidimensional approach that combines pharmacotherapy and psychotherapy (Grade of recommendation: GPP) (Smith et al., 2006; Schroder et al., 2012), and Brief Dynamic Interpersonal Therapy (Grade of recommendation: GPP) (Sattel et al., 2012) have been evaluated.

Finally, studies that have evaluated the impact of cognitive-behavioral therapies on the reduction of chronic abdominal pain in children and adolescents, provide preliminary evidence of its effectiveness (Grade of recommendation: C) (Weydert et al., 2003; Youssef et al., 2004; Groß and Warschburger, 2013).

## The italian consensus conference on pain in neurorehabilitation

The following Authors, who are listed in alphabetical order, contributed to the work of the Italian Consensus Conference on Pain in Neurorehabilitation:

**Michela Agostini**, Neurorehabilitation Department, Foundation IRCCS San Camillo Hospital, Venice, Italy; **Enrico Alfonsi**, C. Mondino National Institute of Neurology Foundation, IRCCS, Pavia, Italy; **Anna Maria Aloisi**, Department of Medicine, Surgery and Neuroscience, University of Siena, Siena, Italy; **Elena Alvisi**, Department of Brain and Behavioural Sciences, University of Pavia, Pavia, Italy; **Irene Aprile**, Don Gnocchi Foundation, Milan, Italy; **Michela Armando**, Department of Neuroscience and Neurorehabilitation, Bambin Gesu' Children's Hospital, IRCCS, Rome, Italy**; Micol Avenali**, C. Mondino National Institute of Neurology Foundation, IRCCS, Pavia, Italy, Department of Brain and Behavioural Sciences, University of Pavia, Pavia, Italy**; Eva Azicnuda**, IRCCS Santa Lucia Foundation, Rome, Italy**; Francesco Barale**, Department of Brain and Behavioural Sciences, University of Pavia, Pavia, Italy**; Michelangelo Bartolo**, Neurorehabilitation Unit, IRCCS INM Neuromed, Pozzilli, Italy**; Roberto Bergamaschi**, C. Mondino National Institute of Neurology Foundation, IRCCS, Pavia, Italy**; Mariangela Berlangieri**, Department of Brain and Behavioural Sciences, University of Pavia, Pavia, Italy**; Vanna Berlincioni**, Department of Brain and Behavioural Sciences, University of Pavia, Pavia, Italy**; Laura Berliocchi**, Department of Health Sciences, University Magna Graecia of Catanzaro, Catanzaro, Italy**; Eliana Berra**, C. Mondino National Institute of Neurology Foundation, IRCCS, Pavia, Italy**; Giulia Berto**, Department of Neurological and Movement Sciences, University of Verona, Verona, Italy**; Silvia Bonadiman**, Department of Neurological and Movement Sciences, University of Verona, Verona, Italy**; Sara Bonazza**, Department of Surgery, University of Verona, Verona, Italy**; Federica Bressi**, Campus Biomedico University, Rome, Italy**; Annalisa Brugnera**, Department of Neurological and Movement Sciences, University of Verona, Verona, Italy**; Stefano Brunelli**, IRCCS Santa Lucia Foundation, Rome, Italy**; Maria Gabriella Buzzi**, IRCCS Santa Lucia Foundation, Rome, Italy**; Carlo Cacciatori**, Department of Neurological and Movement Sciences, University of Verona, Verona, Italy**; Andrea Calvo**, Rita Levi Montalcini Department of Neuroscience, University of Turin, Turin, Italy**; Cristina Cantarella**, Physical and Rehabilitation Medicine Unit, Tor Vergata University, Rome, Italy**; Augusto Caraceni**, Palliative Care, Pain Therapy and Rehabilitation, Fondazione IRCCS Istituto Nazionale dei Tumori di Milano, Milan, Italy**; Roberto Carone**, Neuro-Urology Department, City Hospital Health and Science of the City of Turin, Turin, Italy**; Elena Carraro**, Neuropediatric Rehabilitation Unit, E. Medea Scientific Institute, Conegliano, Italy**; Roberto Casale**, Department of Clinical Neurophysiology and Pain Rehabilitation Unit, Foundation Salvatore Maugeri IRCCS, Montescano, Italy**; Paola Castellazzi**, Department of Neurological and Movement Sciences, University of Verona, Verona, Italy**; Gianluca Castelnuovo**, Psychology Research Laboratory, Istituto Auxologico Italiano IRCCS, Ospedale San Giuseppe, Verbania, Italy, Department of Psychology, Catholic University of Milan, Italy**; Adele Castino**, ASL of the Province of Lodi, Lodi, Italy**; Rosanna Cerbo**, Hub Terapia del Dolore Regione Lazio, Policlinico Umberto I, Sapienza University, Rome Italy**; Adriano Chiò**, Rita Levi Montalcini Department of Neuroscience, University of Turin, Turin, Italy**; Cristina Ciotti**, Physical and Rehabilitation Medicine Unit, Tor Vergata University, Rome, Italy**; Carlo Cisari**, Department of Health Sciences, Università del Piemonte Orientale, Novara, Italy**; Daniele Coraci**, Department of Orthopaedic Science, Sapienza University, Rome, Italy**; Elena Dalla Toffola**, Department of Clinical, Surgical, Diagnostic and Pediatric Sciences, University of Pavia, Pavia, Italy, IRCCS Policlinico San Matteo Foundation, Pavia**; Giovanni Defazio**, Department of Basic Medical Sciences, Neuroscience and Sensory Organs, Aldo Moro University of Bari, Bari, Italy**; Roberto De Icco**, C. Mondino National Institute of Neurology Foundation, IRCCS, Pavia, Italy, Department of Brain and Behavioural Sciences, University of Pavia, Pavia, Italy**; Ubaldo Del Carro**, Section of Clinical Neurophysiology and Neurorehabilitation, San Raffaele Hospital, Milan, Italy**; Andrea Dell'Isola**, Department of Health Sciences, Università del Piemonte Orientale, Novara, Italy**; Antonio De Tanti**, Cardinal Ferrari Rehabilitation Center, Santo Stefano Rehabilitation Institute, Fontanellato, Italy**; Mariagrazia D'Ippolito**, IRCCS Santa Lucia Foundation, Rome, Italy**; Elisa Fazzi**, Childhood and Adolescence Neurology and Psychiatry Unit, City Hospital, Brescia, Italy, Department of Clinical and Experimental Sciences, University of Brescia, Brescia, Italy**; Adriano Ferrari**, Children Rehabilitation Unit, IRCCS Arcispedale S.Maria Nuova, Reggio Emilia, Italy**; Sergio Ferrari**, Department of Neurological and Movement Sciences, University of Verona, Verona, Italy**; Francesco Ferraro**, Section of Neuromotor Rehabilitation, Department of Neuroscience, Azienda Ospedaliera Carlo Poma, Mantova, Italy**; Fabio Formaglio**, Palliative Care, Pain Therapy and Rehabilitation, Fondazione IRCCS Istituto Nazionale dei Tumori di Milano, Milan, Italy**; Rita Formisano**, IRCCS Santa Lucia Foundation, Rome, Italy**; Simone Franzoni**, Poliambulanza Foundation Istituto Ospedaliero, Geriatric Research Group, Brescia, Italy**; Francesca Gajofatto**, Department of Neurological and Movement Sciences, University of Verona, Verona, Italy**; Marialuisa Gandolfi**, Department of Neurological and Movement Sciences, University of Verona, Verona, Italy**; Barbara Gardella**, IRCCS Policlinico San Matteo Foundation, Pavia**; Pierangelo Geppetti**, Department of Health Sciences, Section of Clinical Pharmacology and Oncology, University of Florence, Florence, Italy**; Alessandro Giammò**, Neuro-Urology Department, City Hospital Health and Science of the City of Turin, Turin, Italy**; Raffaele Gimigliano**, Department of Physical and Mental Health, Second University of Naples, Naples, Italy**; Emanuele Maria Giusti**, Department of Psychology, Catholic University of Milan, Italy**; Elena Greco**, Department of Neurological and Movement Sciences, University of Verona, Verona, Italy**; Valentina Ieraci**, Department of Oncology and Neuroscience, University of Turin, City Hospital Health and Science of the City of Turin, Turin, Turin, Italy**; Marco Invernizzi**, Department of Health Sciences, Università del Piemonte Orientale, Novara, Italy**; Marco Jacopetti**, University of Parma, Parma, Italy**; Marco Lacerenza**, Casa di Cura San Pio X S.r.l., HUMANITAS, Milan, Italy**; Silvia La Cesa**, Department of Neurology and Psychiatry, University Sapienza, Rome, Italy**; Davide Lobba**, Department of Neurological and Movement Sciences, University of Verona, Verona, Italy**; Gian Mauro Manzoni**, Psychology Research Laboratory, Istituto Auxologico Italiano IRCCS, Ospedale San Giuseppe, Verbania, Italy, Department of Psychology, Catholic University of Milan, Italy**; Francesca Magrinelli**, Department of Neurological and Movement Sciences, University of Verona, Verona, Italy**; Silvia Mandrini**, Department of Clinical, Surgical, Diagnostic and Pediatric Sciences, University of Pavia, Pavia, Italy**; Umberto Manera**, Rita Levi Montalcini Department of Neuroscience, University of Turin, Turin, Italy**; Paolo Marchettini**, Pain Medicine Center, Hospital San Raffaele, Milan, Italy**; Enrico Marchioni**, C. Mondino National Institute of Neurology Foundation, IRCCS, Pavia, Italy**; Sara Mariotto**, Department of Neurological and Movement Sciences, University of Verona, Verona, Italy**; Andrea Martinuzzi**, Neuropediatric Rehabilitation Unit, E. Medea Scientific Institute, Conegliano, Italy**; Marella Masciullo**, IRCCS Santa Lucia Foundation, Rome, Italy**; Susanna Mezzarobba**, Department of Medicine, Surgery and Health Sciences, University of Trieste, Trieste, Italy**; Danilo Miotti**, Palliative Care and Pain Therapy Unit, Fondazione Salvatore Maugeri IRCCS, Scientific Institute of Pavia, Pavia, Italy**; Angela Modenese**, Department of Neurological and Movement Sciences, University of Verona, Verona, Italy**; Marco Molinari**, IRCCS Santa Lucia Foundation, Rome, Italy**; Salvatore Monaco**, Department of Neurological and Movement Sciences, University of Verona, Verona, Italy**; Giovanni Morone**, IRCCS Santa Lucia Foundation, Rome, Italy**; Rossella Nappi**, Department of Clinical, Surgical, Diagnostic and Pediatric Sciences, University of Pavia, Pavia, Italy, IRCCS Policlinico San Matteo Foundation, Pavia**; Stefano Negrini**, Don Gnocchi Foundation, Milan, Italy, Department of Clinical and Experimental Sciences, University of Brescia, Brescia, Italy**; Andrea Pace**, Neuro-Oncology Unit, Regina Elena National Cancer Institute of Rome, Rome, Italy**; Luca Padua**, Don Gnocchi Foundation, Milan, Italy, Institute of Neurology, Catholic University, Rome, Italy**; Emanuela Pagliano**, Developmental Neurology Unit, C. Besta Neurological Institute Foundation, Milan, Italy**; Valerio Palmerini**, Hub Terapia del Dolore Regione Lazio, Policlinico Umberto I, Sapienza University, Rome Italy**; Stefano Paolucci**, IRCCS Santa Lucia Foundation, Rome, Italy**; Costanza Pazzaglia**, Don Gnocchi Foundation, Milan, Italy**; Cristiano Pecchioli**, Don Gnocchi Foundation, Milan, Italy**; Alessandro Picelli**, Department of Neurological and Movement Sciences, University of Verona, Verona, Italy**; Carlo Adolfo Porro**, Department of Biomedical, Metabolic and Neural Sciences, University of Modena and Reggio Emilia, Modena, Italy**; Daniele Porru**, IRCCS Policlinico San Matteo Foundation, Pavia**; Marcello Romano**, Neurology Unit, Azienda Ospedaliera Ospedali Riuniti Villa Sofia Cervello, Palermo, Italy**; Laura Roncari**, Department of Neurological and Movement Sciences, University of Verona, Verona, Italy**; Riccardo Rosa**, Hub Terapia del Dolore Regione Lazio, Policlinico Umberto I, Sapienza University, Rome Italy**; Marsilio Saccavini**, ASL 2 Bassa Friulana-Isontina, Italy**; Paola Sacerdote**, Department of Pharmacological and Biomolecular Sciences, University of Milano, Milano, Italy**; Giorgio Sandrini**, C. Mondino National Institute of Neurology Foundation, IRCCS, Pavia, Italy, Department of Brain and Behavioural Sciences, University of Pavia, Pavia, Italy**; Donatella Saviola**, Cardinal Ferrari Rehabilitation Center, Santo Stefano Rehabilitation Institute, Fontanellato, Italy**; Angelo Schenone**, Department of Neuroscience, Rehabilitation, Ophthalmology, Genetics, Maternal and Child Health (DiNOGMI), University of Genoa, Genoa, Italy**; Vittorio Schweiger**, Department of Surgery, University of Verona, Verona, Italy**; Giorgio Scivoletto**, IRCCS Santa Lucia Foundation, Rome, Italy**; Nicola Smania**, Department of Neurological and Movement Sciences, University of Verona, Verona, Italy**; Claudio Solaro**, Neurology Unit, ASL3, Genoa, Italy**; Vincenza Spallone**, Department of Systems Medicine, University Tor Vergata, Rome, Italy**; Isabella Springhetti**, Functional Recovery and Rehabilitation Unit, IRCCS Fondazione S. Maugeri, Pavia, Italy**; Stefano Tamburin**, Department of Neurological and Movement Sciences, University of Verona, Verona, Italy**; Cristina Tassorelli**, C. Mondino National Institute of Neurology Foundation, IRCCS, Pavia, Italy, Department of Brain and Behavioural Sciences, University of Pavia, Pavia, Italy**; Michele Tinazzi**, Department of Neurological and Movement Sciences, University of Verona, Verona, Italy**; Rossella Togni**, Department of Clinical, Surgical, Diagnostic and Pediatric Sciences, University of Pavia, Pavia, Italy**; Monica Torre**, IRCCS Santa Lucia Foundation, Rome, Italy**; Riccardo Torta**, Department of Oncology and Neuroscience, University of Turin, City Hospital Health and Science of the City of Turin, Turin, Turin, Italy**; Marco Traballesi**, IRCCS Santa Lucia Foundation, Rome, Italy**; Marco Tramontano**, IRCCS Santa Lucia Foundation, Rome, Italy**; Andrea Truini**, Department of Neurology and Psychiatry, University Sapienza, Rome, Italy**; Valeria Tugnoli**, Neurological Unit, University Hospital of Ferrara, Ferrara, Italy**; Andrea Turolla**, Neurorehabilitation Department, Foundation IRCCS San Camillo Hospital, Venice, Italy**; Gabriella Vallies**, Department of Neurological and Movement Sciences, University of Verona, Verona, Italy**; Elisabetta Verzini**, Department of Neurological and Movement Sciences, University of Verona, Verona, Italy**; Mario Vottero**, Neuro-Urology Department, City Hospital Health and Science of the City of Turin, Turin, Italy**; Paolo Zerbinati**, Neuro-orthopaedic Program, Hand Surgery Department, Santa Maria Hospital MultiMedica, Castellanza, Italy.

## Author contributions

All authors listed, have made substantial, direct and intellectual contribution to the work, and approved it for publication.

### Conflict of interest statement

The authors declare that the research was conducted in the absence of any commercial or financial relationships that could be construed as a potential conflict of interest.
